# Optimal sensor set for MEG-based spontaneous and intended speech decoding toward practical communication

**DOI:** 10.3389/fnhum.2026.1925703

**Published:** 2026-09-23

**Authors:** Pedro Andres Alba Diaz, Jinuk Kwon, Karinne Berstis, Matthew Weed, Paul Ferrari, Jun Wang

**Affiliations:** 1 Department of Speech, Language, and Hearing Sciences, The University of Texas at Austin, Austin, TX, United States; 2 Department of Neurology, The University of Texas at Austin, Austin, TX, United States; 3 MyndVox Inc., Austin, TX, United States; 4 Jack H. Miller Magnetoencephalography (MEG) Center, Helen DeVos Children's Hospital, Grand Rapids, MI, United States; 5 Department of Pediatrics and Human Development, Michigan State University, Lansing, MI, United States

**Keywords:** brain-computer interfaces, magnetoencephalography, neural speech decoding, NV-diamond, OPM

## Abstract

**Introduction:**

Neural speech decoding has demonstrated great potential as an alternative to current brain-computer interface (BCI) spellers, enabling a faster, more natural way of communication for patients who have lost voluntary abilities, including speech. Most neural speech decoding studies have focused on decoding cued speech, in which participants are presented with visual or auditory signals before producing imagined or overt speech. Only a few recent studies have investigated the decoding of spontaneous and intended speech without external cues using neural activity acquired from a full-scalp clinical magnetoencephalography (MEG) system. With recent advances in MEG technology, including optically pumped magnetometers (OPMs) and nitrogen-vacancy (NV) center (NV-diamond) magnetometers, MEG is emerging as a next-generation wearable system for practical speech BCIs. However, it remains unclear how many sensors are needed, where to place them, and what sampling frequency is optimal for wearable MEG devices that require cost-effective, computationally efficient operation.

**Methods:**

In this study, we addressed these gaps by using a forward selection algorithm to find an optimal subset of sensors for spontaneous intended and overt speech decoding and by evaluating decoding performance across different frequency bands. Support vector machine and random forest were used as the classifiers in these experiments.

**Results:**

Our results indicate that only a small number of sensors (7–8 on average, with a maximum of fewer than 24) are sufficient to achieve decoding performance comparable to that obtained using the whole scalp (more than 200) sensors. In addition, we observed that the Lower High-Gamma (62–125 Hz) band yielded the best performance in some settings, although not statistically significantly higher than other bands in all settings.

**Discussion:**

These findings suggest that future wearable MEG devices may not need the whole-scalp sensor set and should support a sampling rate of at least 250 Hz, while 500 Hz is also encouraged, if possible. In summary, these findings provide practical insights for MEG-based BCIs for communication.

## 1 Introduction

Locked-in syndrome is a devastating condition that frequently appears in severe brain damage or advanced stages of neurodegenerative diseases such as amyotrophic lateral sclerosis (ALS), in which people retain consciousness and cognitive function, but lose nearly all voluntary motor abilities, including speech (Laureys et al., 2005; Smith and Delargy, 2005). Although limited movements (e.g., vertical eye shifts or eyelid motion) can sometimes allow very basic signaling, patients who are completely locked-in lack any reliable motor pathway for communication (Smith and Delargy, 2005; Kohnen et al., 2013). For these patients, augmentative and alternative communication (AAC) systems that bypass the damaged motor system represent their only option for communication.

Brain-computer interfaces (BCIs) provide a way to create such a communication channel directly from neural signals. So far, the most commonly used BCIs for this goal are electroencephalography (EEG)-based speller systems, which enable users to choose letters or symbols by generating evoked or modulated brain responses (Rezeika et al., 2018). Although these systems have proven useful in real-world settings, their communication speed is inherently constrained: users must spell character by character, producing rates far below natural conversational speed and placing substantial visual and cognitive fatigue and temporal demands on the user (Kundu and Ari, 2022; Rezeika et al., 2018). This gap between achievable and natural communication rates remains the main barrier to clinically satisfactory AAC.

Neural speech decoding has emerged as a promising alternative that addresses these limitations of BCI spellers. Instead of reconstructing speech by one letter/character at a time, mapping neural signals directly to speech or text offers the possibility of restoring communication with speed and naturalness similar to everyday conversation (Anumanchipalli et al., 2019; Iljina et al., 2017). In recent work, researchers have decoded entire sentences and continuous question-and-answer exchanges from cortical activity and have used these signals to control speech synthesizers and text-based interfaces (Anumanchipalli et al., 2019; Moses et al., 2019, 2021; Dash et al., 2020a). Together, these findings suggest that decoding speech itself, rather than relying on intermediary spelling-based tasks, constitutes a more direct route to achieving naturalistic communication for individuals with profound motor paralysis, such as patients with locked-in syndrome.

To date, the most effective demonstrations of directly translating neural activity into speech or text have been based on invasive brain recordings. For example, Card et al. (2024) showed that intracortical microelectrode arrays can be used to rapidly calibrate speech neuroprostheses with only 30 mins of cortical recordings. In an individual with ALS, their approach decoded intended speech with an accuracy of 90.2% from a vocabulary of 125,000 words, reaching performance levels sufficient to support conversational communication after only a short training period (Card et al., 2024). Similarly, electrocorticography (ECoG)—which measures field potentials from the cortical surface—has supported the decoding of phonemes and words, as well as the characterization of speech-related cortical regions (Derix et al., 2014; Herff and Schultz, 2016; Kanas et al., 2014; Ramsey et al., 2018; Song et al., 2014; Towle et al., 2008). However, despite their impressive performance, all of these techniques require neurosurgery to place electrodes on the brain's surface or within its tissue. In addition, the signal-to-noise ratio of the signals recorded by these implanted electrodes deteriorates over time (Vansteensel et al., 2024; Hughes et al., 2021; Barrese et al., 2013). This invasiveness introduces surgical risks and can discourage many prospective users, thereby limiting their scalability as widely accessible communication tools.

In light of this, non-invasive neuroimaging presents an alternative approach and has consequently attracted increasing attention to speech decoding. Techniques such as functional magnetic resonance imaging (fMRI) and functional near-infrared spectroscopy (fNIRS) indirectly infer neural activity from changes in blood oxygenation (Ferrari and Quaresima, 2012). Although these methods are informative and suitable for semantic decoding (Tang et al., 2023), their temporal resolution—on the order of seconds—is too low for speech. fNIRS is further constrained by low spatial resolution and shallow recording depth (Scarapicchia et al., 2017). Other noninvasive approaches include electroencephalography (EEG) and magnetoencephalography (MEG), which directly record the electric and magnetic fields generated by postsynaptic neuronal currents, capturing brain dynamics in real-time. Although their precision still falls short of invasive recordings, EEG/MEG have the greatest potential for this application and have consistently enabled the classification of closed-set vocabularies of phrases Dash et al., (2020a, 2021a), words (D'Zmura et al., 2009; Garcia-Salinas et al., 2019; Panachakel and Ramakrishnan, 2021), and phonemes (Chi et al., 2011; Sakai et al., 2021; Suyuncheva et al., 2021), thus demonstrating the promise of noninvasive speech-BCIs.

Among noninvasive recording methods, MEG offers several unique strengths for speech decoding. In contrast to the electrical potentials captured by EEG, the magnetic fields recorded by MEG pass through the dura, skull, and scalp with little distortion, providing a more accurate reflection of the underlying neural generators and producing a higher spatial resolution than EEG or fNIRS, while also maintaining millisecond temporal resolution —far surpassing that of fMRI (Cohen and Cuffin, 1983). In line with these properties, MEG studies have characterized the rapid temporal dynamics of speech production and speech-evoked articulatory processes, underscoring the utility of neuromagnetic signals for speech decoding (Dash et al., 2020b, 2021a; Memarian et al., 2012). However, translating MEG into real-world clinical AAC applications has so far been constrained by the device itself. Conventional MEG devices are based on superconducting quantum interference devices (SQUIDs), which require cryogenic cooling, a large, rigid, and immobile helmet, and a carefully maintained magnetically shielded room. As a result, these systems are costly, non-portable, and impractical for home use.

Emerging magnetic field sensing technologies, particularly optically pumped magnetometers (OPMs) and nitrogen-vacancy (NV) center (NV-diamond) magnetometers, offer promising solutions to these limitations. Unlike SQUIDs, OPMs operate near room temperature and can be placed directly on the scalp. The resulting reduction in source-to-sensor distance produces signal amplitudes roughly four times higher than those obtained with conventional scalp-distant SQUIDs (Boto et al., 2017). As a consequence, OPM can reach a sensitivity of 1–10 fTHz^−1/2^, competitive with SQUID-based MEG. And, because each unit is small and independently mounted, OPMs can be arranged into lightweight, movement-tolerant arrays adapted to an individual head (Boto et al., 2018; Roberts et al., 2019). These advantages have been demonstrated for tasks directly relevant to speech-BCIs: a wearable array of 45–46 OPMs in a 3D-printed helmet recovered the cortical tracking of continuous speech (de Lange et al., 2021), and a compact 24-channel OPM system provided higher classification accuracies than SQUIDs in a single-trial classification of evoked responses to auditory tones (Iivanainen et al., 2023). In parallel, NV-diamond magnetometers using nitrogen-vacancy (NV) centers are advancing toward ambient, no-magnetic-shielding infrastructure, room-temperature operation with millimeter-scale spatial resolution. Although a higher level of sensitivity level is needed, a recent progress in diamond-based system achieved a sensitivity of 9.4 ± 0.1 pTHz^−1/2^ in the near-DC 5–100 Hz band that spans clinically relevant neuromagnetic activity, and its sensor head can be positioned within ~1 mm of the target, reducing signal loss associated with source-to-sensor separation (Sekiguchi et al., 2024). Together, these advances reduce infrastructure barriers and bring wearable MEG-based speech-BCIs closer to practical clinical deployment.

Designing such a wearable MEG system for speech communication demands more than simply having suitable sensors available: it requires a precise understanding of what a wearable sensor montage must measure to decode speech reliably. The size and infrastructure cost of SQUID-based systems are not heavily impacted by the sensor count, and so hundreds of fixed channels are the standard device design. For the wearable technologies, the opportunity to assemble small numbers of sensor nodes whose placement and acquisition bandwidth are design variables will directly influence cost, portability, and ease of use—the last being a major utility bottleneck for ACC users. However, the sensing requirements for speech decoding have not been systematically defined. Previous work has shown that speech decoding performance can be largely preserved using a reduced subset of MEG sensors (Dash et al., 2020c), indicating that full-scalp coverage may not be essential, at least for cued (imagined or overt) speech tasks, on which this study was based.

In real-world communication, speech is rarely prompted by external cues. For a speech-BCI to be clinically useful, it must decode speech that users initiate spontaneously. This scenario is more representative of everyday communication and more challenging because it lacks the stimulus-locked neural responses that many cued paradigms rely on. This distinction is also important from a decoding perspective. In cue-based tasks, neural activity related to speech production can be difficult to separate from neural responses evoked by the cue itself. In contrast, spontaneous speech more cleanly isolates the speech-related neural signals that the BCI aims to decode. Dash et al. (2024) demonstrated that this can be achieved non-invasively, decoding spontaneous speech from MEG signals at accuracies above chance level. However, it remains unknown whether the sensor reduction demonstrated for cue-based speech tasks (Dash et al., 2020c) extends to this more realistic setting, and if so, how wearable systems should be configured to achieve it. In addition, the frequency content required for reliable spontaneous speech decoding has not been systematically examined, despite its importance for determining the acquisition bandwidth and sampling rate of wearable MEG systems.

We address this question by defining the MEG sensor specifications required for next-generation wearable speech-BCI decoders operating on spontaneous speech. Using simultaneously recorded MEG and speech data, we pursue three aims: (1) determine the minimum number of sensors that preserve spontaneous speech decoding accuracy; (2) identify the optimal scalp locations of those sensors; and (3) determine the frequency band that contributes most to speech decoding, thereby informing the minimum sampling rate required for wearable MEG systems. By establishing these requirements for both spontaneous and intended speech, we provide design parameters to guide the design of the next-generation wearable MEG-based systems toward practical applications.

## 2 Materials and methods

### 2.1 Dataset and participants

The whole dataset collected in Dash et al. (2024) was reused in this study. Neuromagnetic and acoustic data were recorded from seven healthy adult English speakers (five females; aged 22–30 years) who reported no known neurological, speech, language, hearing, or cognitive impairments. The participants were seated and kindly asked to randomly pronounce the words *yes* and *no* every 4–5 s, without any external cue or prompt. The absence of cues ensured that the recorded neuromagnetic signals reflected speech-motor and premotor processes rather than stimulus-evoked responses. Each participant provided approximately 80 repetitions for each word, without strict balancing and without necessarily alternating between yes and no responses.

### 2.2 MEG data acquisition

Neuromagnetic brain activity (Figure 1A) was recorded using a 306–channel (204 gradiometers, 102 magnetometers) MEGIN TRIUX system (MEGIN LLC, FL, USA). The signals were sampled at 4 kHz using an online hardware band-pass filter ranging from 0.03 to 1,333 Hz. The audio was recorded and time-locked to the MEG acquisition to capture spoken words. Two sessions per participant were recorded. For decoding, only gradiometer channels were used, in line with previous findings showing that gradiometers provide more informative features than magnetometers for MEG-based speech decoding (Dash et al., 2021a).

**Figure 1:**
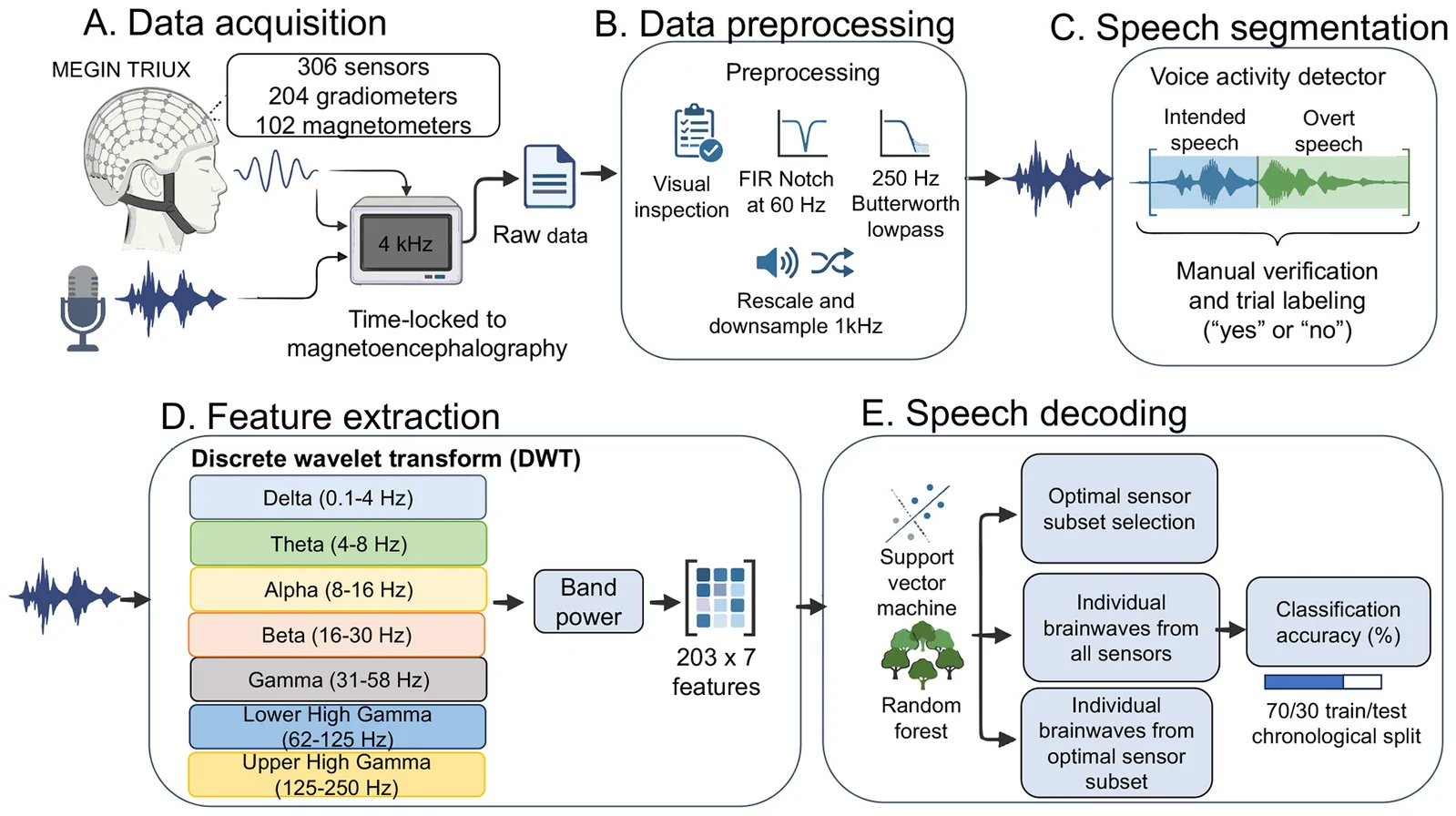
Overview of the magnetoencephalography (MEG) speech decoding pipeline. **(A)** Data acquisition: raw MEG recorded at 4 kHz with a MEGIN TRIUX system (306 sensors: 204 gradiometers, 102 magnetometers) and time-locked audio. **(B)** Preprocessing: visual inspection, 60 Hz (and harmonic) notch filtering, 250 Hz Butterworth low-pass filtering, and downsampling to 1 kHz. **(C)** Speech segmentation: voice activity detection to identify overt speech, followed by manual verification and binary trial labeling (*yes*/*no*); overt and intended speech share the same duration. **(D)** Feature extraction: Discrete Wavelet Transform decomposition into seven bands—Delta (0.1–4 Hz), Theta (4–8 Hz), Alpha (8–16 Hz), Beta (16–30 Hz), Gamma (31–58 Hz), Lower High-Gamma (62–125 Hz), and Upper High-Gamma (125–250 Hz)—yielding a 203 × 7 bandpower feature matrix. **(E)** Speech decoding: Support Vector Machine and Random Forest classifiers evaluate accuracy using a 70/30 chronological train/test split across three decoding analyses, including optimal sensor selection and decoding with individual bands from all sensors and from optimal sensor subsets. Created in BioRender. ALBA DIAZ, P. A. (2026) https://BioRender.com/bv6xijy.

### 2.3 Data preprocessing

During preprocessing (Figure 1B), visual inspection of the power spectral density among participants revealed a flat gradiometer channel, which was later excluded, leaving 203 gradiometers for use. Power line interference was suppressed by applying finite-impulse-response (FIR) Notch filters at 60 Hz and its harmonics. Then a fourth-order Butterworth infinite-impulse-response (IIR) low-pass filter was applied with a cutoff frequency of 250 Hz. Finally, the recordings were downsampled from 4 kHz to 1 kHz, and the audio samples at event onset were rescaled accordingly.

### 2.4 Speech segmentation

Speech onset and offset times were derived from the synchronously acquired audio channel using a voice activity detector (VAD). Acoustic labels were manually verified by listening to the recording, and each trial was assigned to the *yes* or *no* class. Then each trial was segmented into two equal-duration speech segments relative to acoustic onset and offset, as shown in Figure 1C and in more detail in Figure 2. **Intended speech** was defined as the interval [onset−*d*, onset], where *d* is the *yes*/*no* trial duration. **Overt speech** was defined as [onset, onset+*d*], which corresponds to the duration of the spoken word. Trials with durations lower than 128 samples (128 ms at 1 kHz) and trials in which the phase exceeded the recording boundaries were not included, ensuring that there was no overlap between these trials.

**Figure 2:**
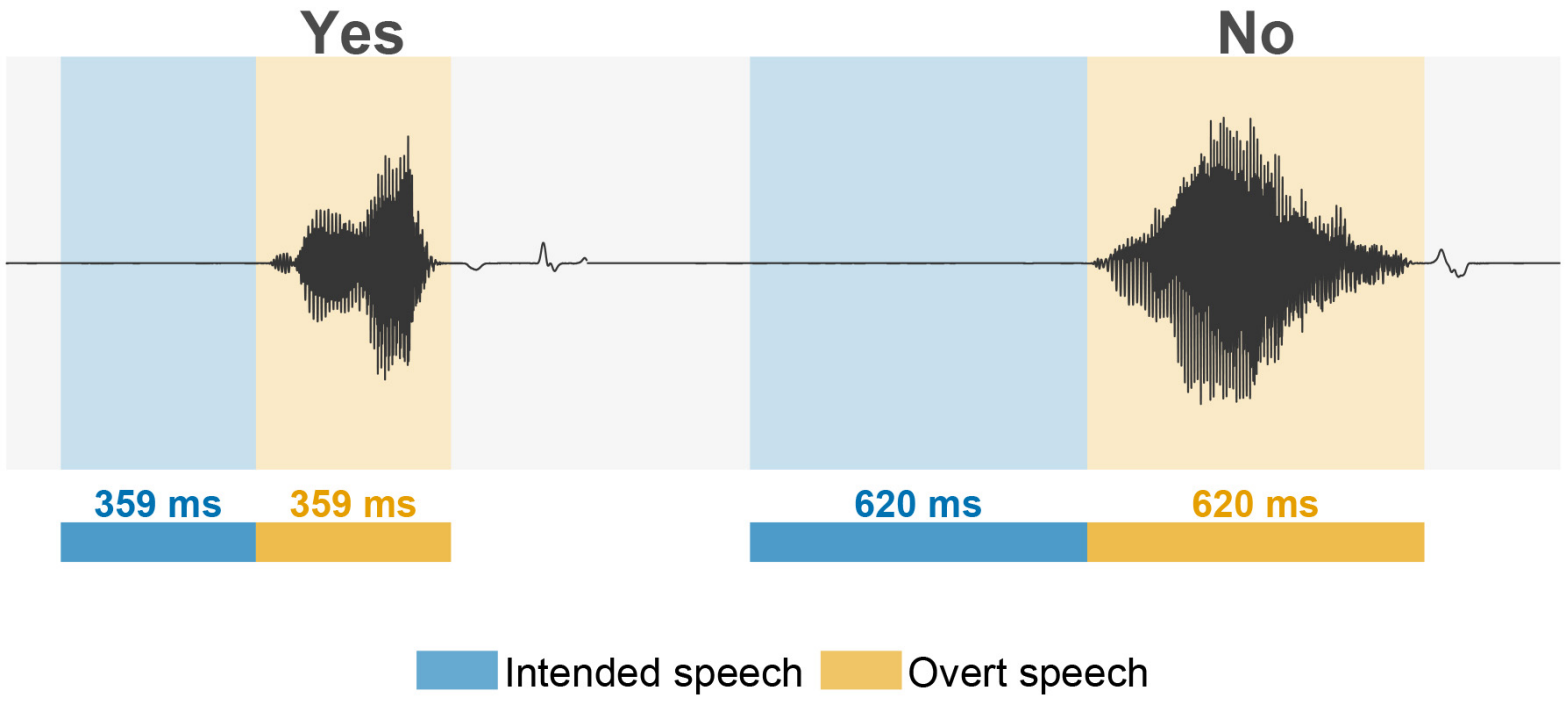
Temporal segmentation of representative *yes* **(left)** and *no* **(right)** trials. Blue: intended speech segment (pre-speech interval). Orange: overt speech segment (acoustic onset to offset). Orange borders mark acoustic onsets and offsets as detected by the voice activity detector.

Because there is a lack of consistent definitions of many neural speech decoding terms in the literature, here we define our terminology for this study. Spontaneous speech means no prompt or cue in the stimuli. In contrast, cued speech is preceded by a stimulus, such as a visual or auditory cue. This is the most common behavioral paradigm seen in the literature. Intended speech means the intention before the actual overt speech, which may include the neurological process for generating the intention (to say something) and planning (how to say). Overt speech involves articulatory movement and out-loud production of the speech sounds. In this study we focused on two speech modalities: (1) spontaneous, intended speech, and (2) spontaneous overt speech. The goals are to decode spontaneous intended speech, where spontaneous overt speech served as a reference. For clarity we do not assume our study addressed imagined speech, as to our understanding that is to “think” about speaking without actual articulation (Dash et al., 2020a).

The decoding paradigm in this manuscript is to match the whole word pattern in Figure 2 (classification of the spontaneously produced words yes/no, either intended or overt), rather than phone classes or sentence-levels.

### 2.5 Feature extraction

Spectral-band power (Figure 1D) was extracted using seven-level discrete wavelet transform (DWT) with a Daubechies-4 (db4) mother wavelet decomposed to seven levels (Dash et al., 2018). For each channel, the DWT was applied along the time axis, and the corresponding level's coefficients were used to reconstruct the signal component for each sub-band that corresponds to the conventional Delta, Theta, Alpha, Beta, Gamma, Lower High-Gamma, and Upper High-Gamma frequency ranges. The mean squared amplitude of each reconstructed component was taken as the band power. This produced a feature array (*N*_trials_, 203 channels, 7 bands) for each speech decoding segment. The seven sub-bands and their frequency ranges at a 1 kHz sampling rate were: Delta (0.1–4 Hz), Theta (4–8 Hz), Alpha (8–16 Hz), Beta (16–30 Hz), Gamma (31–58 Hz), Lower High-Gamma (62–125 Hz), and Upper High-Gamma (125–250 Hz). We followed the tradition of these bands in cognitive neuroscience, where Gamma is 31–58 Hz, and High-Gamma is 62–250 Hz. We followed our previous work to further divide High-Gamma into small bands, Lower High-Gamma and Upper High-Gamma (Dash et al., 2021b).

The brainwave band-power values were converted to decibels using 10·log_10_(*P*+ε), and ε = 10^−12^ was added to avoid evaluating log_10_(0). The resulting channel-by-band matrix was flattened into a 1,421-dimensional feature vector (203 channels × 7 bands), which served as the input for speech decoding methods.

### 2.6 Speech decoding

All 203 gradiometers and all seven DWT-derived brainwave features were concatenated into a single feature vector per trial to define a baseline condition. For each speech segment (intended and overt speech), we then performed three separate decoding analyses: (1) optimal sensor subset selection, (2) speech decoding using individual brainwave components across all sensors, and (3) speech decoding using individual brainwave components restricted to a reduced sensor montage, as shown schematically in Figure 1E.

Two widely used machine learning classifiers were used for the analyses: a support vector machine (SVM) with a radial basis function (RBF) kernel and a random forest (RF) ensemble of 500 decision trees. SVM and RF were selected over other commonly used approaches including deep neural networks and variants, due to their rapid execution and solid performance in similar neural speech decoding tasks (Panachakel and Ramakrishnan, 2021; Haresh et al., 2025; Beach et al., 2021). Particularly rapid execution is critical as they will be executed many times during the sensor selection experiment. SVM obtained great performance in our previous sensor selection for imagined and overt speech study using MEG (Dash et al., 2020c). In addition, we employed two distinct machine learning models (rather than just one) to mitigate the risk of model-specific bias, lending greater credibility to the generalization of our findings. For both approaches, features were z-score standardized (fit strictly to the training set), and class weights were balanced to account for any *yes*/*no* trial imbalance. All experiments were performed with a fixed random seed (42) to ensure reproducibility.

All classifiers and decoding analyses were evaluated using a chronological 70/30 hold-out split: the first 70% of each participant's trials were used as the training set and the remaining 30% as the test set. This approach preserves the temporal structure of the MEG data, avoids data leakage, and can be applied to online decoding. The decoding performance is reported as binary *yes*/*no* classification accuracy (%).

Our focus was on decoding intended speech, but we decoded both intended and overt speech, with overt speech as a reference.

All computational processing and analyses were implemented in Python 3 using MNE-Python (Gramfort et al., 2013), scikit-learn (Pedregosa et al., 2011), and PyWavelets (Lee et al., 2019) libraries. The computationally intensive steps were accelerated using the NVIDIA A100-SXM4-80GB GPU (CUDA 13.0).

#### 2.6.1 Optimal sensor subset selection

To determine the minimal sensor configuration that maintains speech decoding accuracy, we implemented a widely used forward sensor selection algorithm (Algorithm 1), adapted from Dash et al. (2020c). The algorithm was run separately for each participant, speech segment, and classifier using only the training set. Beginning with an empty set, it iteratively adds the single gradiometer that maximizes hold-out accuracy when combined with the sensors already selected. Selection stops when no candidate sensor improves accuracy by at least δ = 0.5% for *p* = 3 consecutive iterations, or once *S*_max_ = 100 sensors have been chosen.

Algorithm 1 Forward selection. 
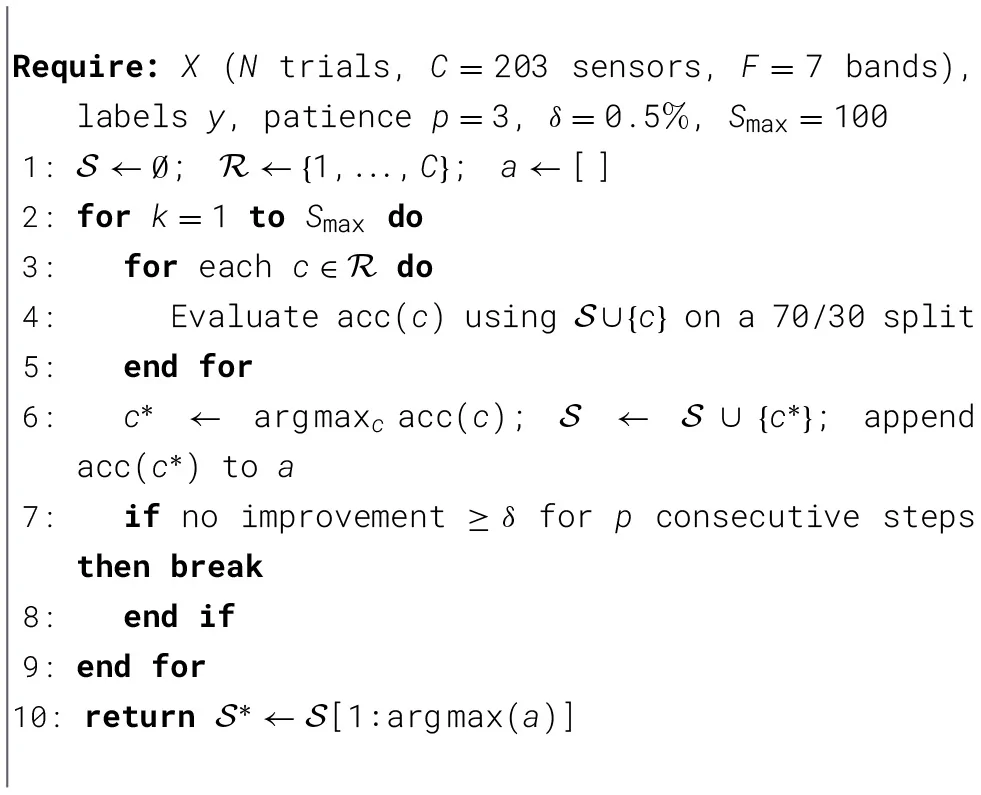

#### 2.6.2 Speech decoding using individual brainwaves from all sensors

Each of the seven DWT-derived frequency bands was independently decoded using all 203 gradiometers. For each band, logarithmic power features were extracted only from that band, resulting in a 203-dimensional feature vector per trial. The SVM and RF classifiers were then trained and evaluated on the same chronological 70/30 hold-out split described above. This analysis assesses whether individual brain waves contain characteristic and distinguishable information about intended and overt speech. In addition, it indirectly identifies the frequency ranges to which emerging magnetic field sensing technologies need to be more sensitive.

#### 2.6.3 Speech decoding using individual brainwaves from the optimal sensor subset

To examine whether the sensor reduction extends across frequency bands, we repeated the frequency-specific decoding using only the optimal sensor subset, $S^{*}$, derived in Section 2.6.1. For each participant, sensors selected via SVM or RF-based forward selection were assigned to the corresponding decoders. As a result, decoding was performed on a reduced feature vector whose dimensionality was tailored to the participant and the speech segment. By contrasting these results with the full-sensor baseline, we can determine whether a sparse, wearable montage maintains the frequency-resolved decoding pattern or whether some brainwaves require more extensive scalp coverage to remain informative.

## 3 Results

Seven participants contributed a total of 1,242 trials (596 *yes*, 646 *no*), corresponding to an average of 86 *yes* and 93 *no* utterances per participant. Trial duration was statistically equivalent between *yes* (mean: 0.37 s) and *no* (mean: 0.52 s) utterances (equivalence test: *p* < 0.05, effect size = 0.16), indicating that duration differences did not confound the decoding results.

As a baseline, both classifiers were first evaluated using all 203 gradiometers and all seven DWT bands (Figure 3), providing the full-montage reference for the reduced-sensor and individual brainwave configurations examined in the following subsections. The two classifiers obtained comparable and above-chance level accuracies for both intended and overt speech. SVM obtained an average accuracy of 61.9%±13.1% for intended speech and 65.7%±13.8% for overt speech (Figure 3A). Random Forest yielded an average accuracy of 64.9%±13.0% for intended speech and 71.0%±13.3% for overt speech (Figure 3B). On average, overt speech decoding accuracies are slightly higher than those from intended speech.

**Figure 3:**
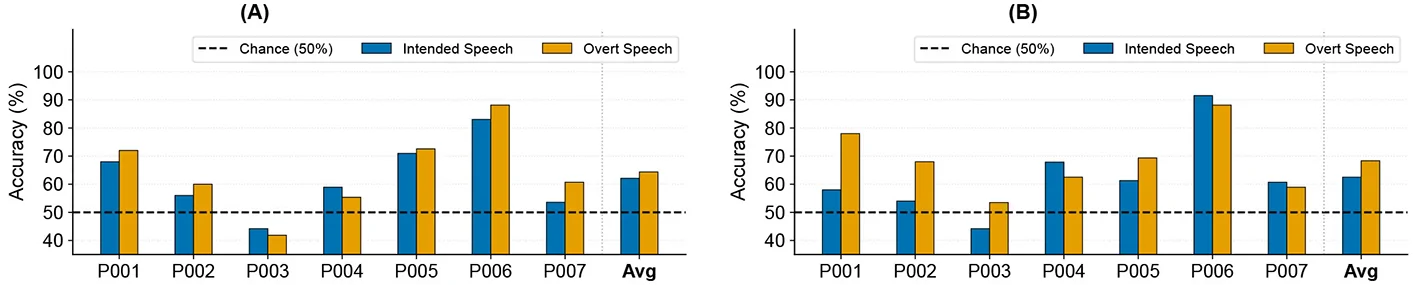
Baseline speech decoding accuracy (%) using all 203 gradiometers and all seven combined DWT bands [**(A)** support vector machine; **(B)** random forest]. Bars indicate accuracy for individual participants and the group mean; colors distinguish speech segment (intended and overt speech). The dashed line denotes the classification chance level (50%).

### 3.1 Optimal sensor subset selection

For each participant, speech segment, and classifier, the forward sensor selection algorithm described in Section 2.6.1 (Algorithm 1) was applied to identify an optimal sensor subset $S^{*}$, defined as the subset that maximized hold-out accuracy before satisfying the patience criterion. The following results are based on these optimized configurations.

#### 3.1.1 Intended speech

Results for the forward selection algorithm are shown in Figure 4. For intended speech, the SVM-based sensor selection converged on an average of 7.4 ± 4.6 sensors per participant (range: 3–16), yielding a mean hold-out decoding accuracy of 85.8%, compared to 62.1% with all 203 sensors. The RF-based selection converged on an average of 11.0 ± 5.4 sensors (range: 4–21), reaching a mean hold-out accuracy of 88.5%, compared to 62.5% with all 203 sensors. These findings indicate that a substantially reduced sensor montage yielded similar to or higher than the performance with the whole-scalp decoding accuracy for intended speech. The scalp distribution of the selected sensors is shown in Figure 5.

**Figure 4:**
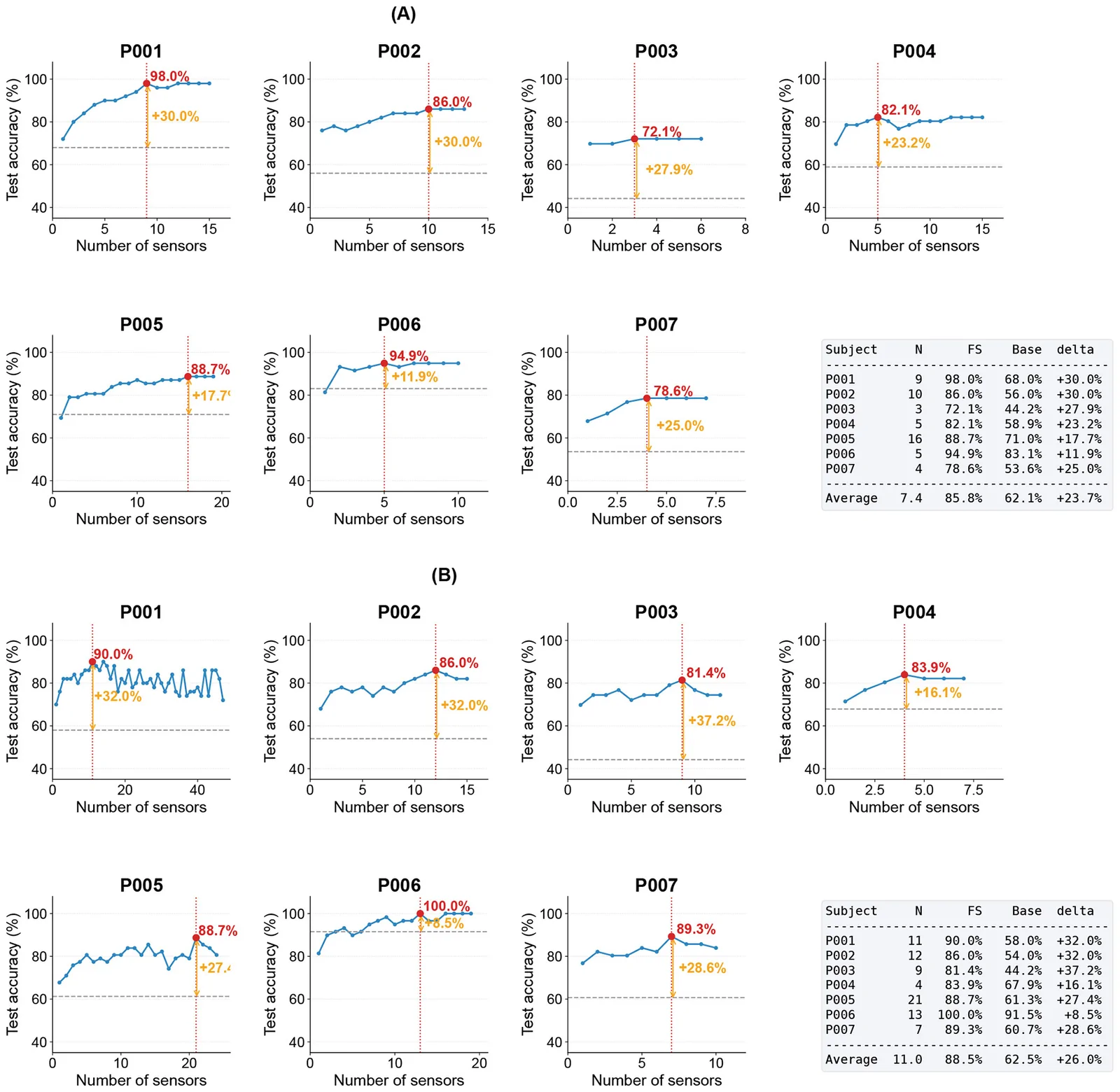
Forward sensor selection accuracy curves for **intended speech** [**(A)** support vector machine; **(B)** random forest]. Hold-out decoding accuracy is shown as a function of the number of sensors iteratively added by Algorithm 1, separately for each participant. The dashed horizontal line indicates the baseline accuracy obtained using all 203 sensors, and the vertical dotted line denotes the optimal sensor count $S^{*}$ at which accuracy peaks.

**Figure 5:**
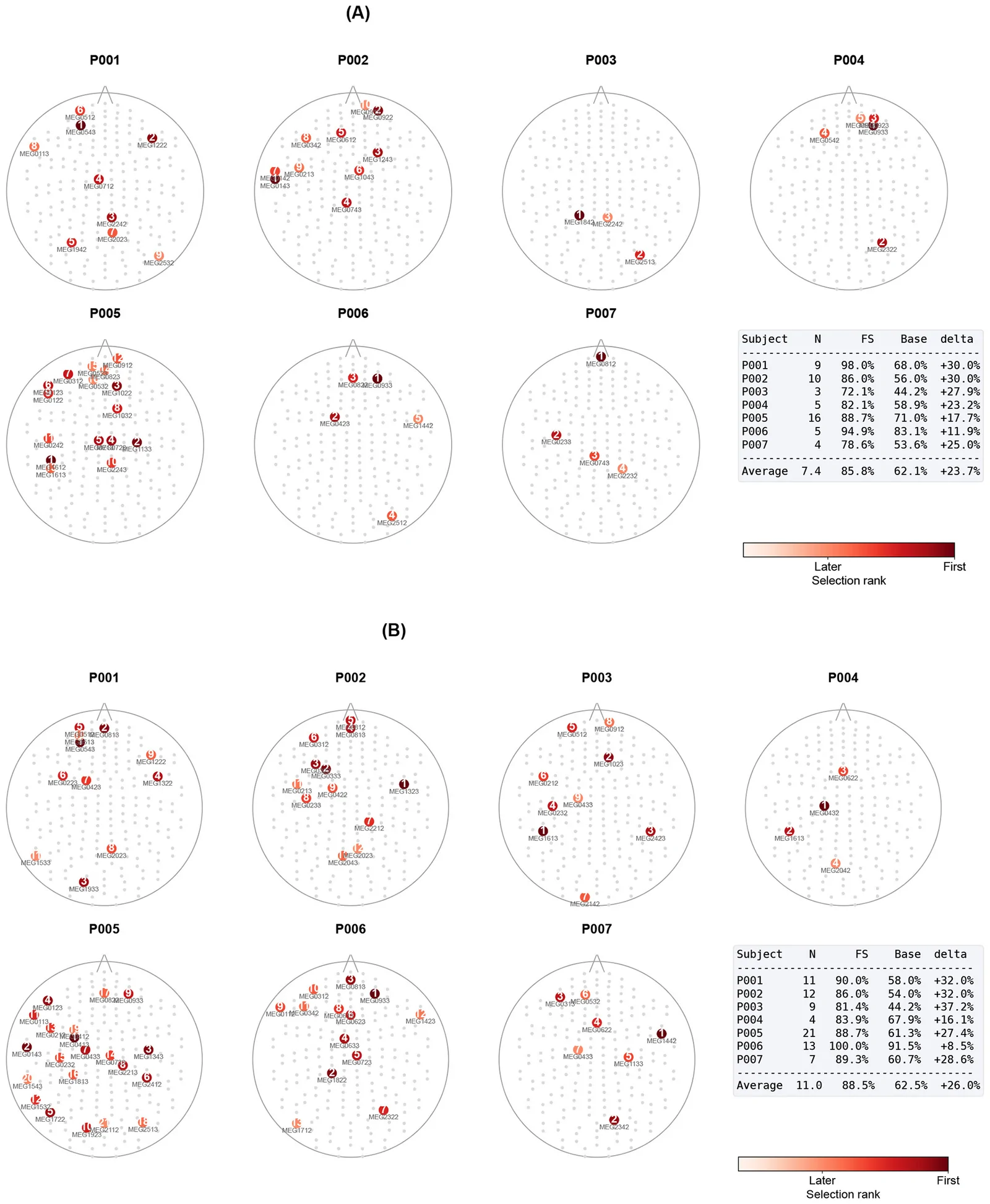
Optimal sensor locations for **intended speech** decoding [**(A)** support vector machine, **(B)** random forest]. Scalp topographies display each participant's optimal sensor subset $S^{*}$. Color denotes selection order (darker red, earlier selection; numbers indicate rank). Gray dots indicate the complete 203-channel montage.

#### 3.1.2 Overt speech

For overt speech, SVM-based sensor selection converged on an average of 6.9 ± 2.6 sensors per participant (range: 3–10), yielding a mean hold-out decoding accuracy of 91.1%, markedly higher than the 64.4% obtained when using all 203 sensors (Figure 6A). RF-based selection converged on an average of 9.3 ± 7.7 sensors (range: 3–22), achieving a mean hold-out accuracy of 91.4%, compared to 68.3% with all 203 sensors (Figure 6B). Relative to intended speech, overt speech decoding relied on a similar number of sensors but attained higher absolute accuracy, consistent with the richer speech-motor information available during active articulation. The scalp distribution of sensors selected for overt speech is shown in Figure 7.

**Figure 6:**
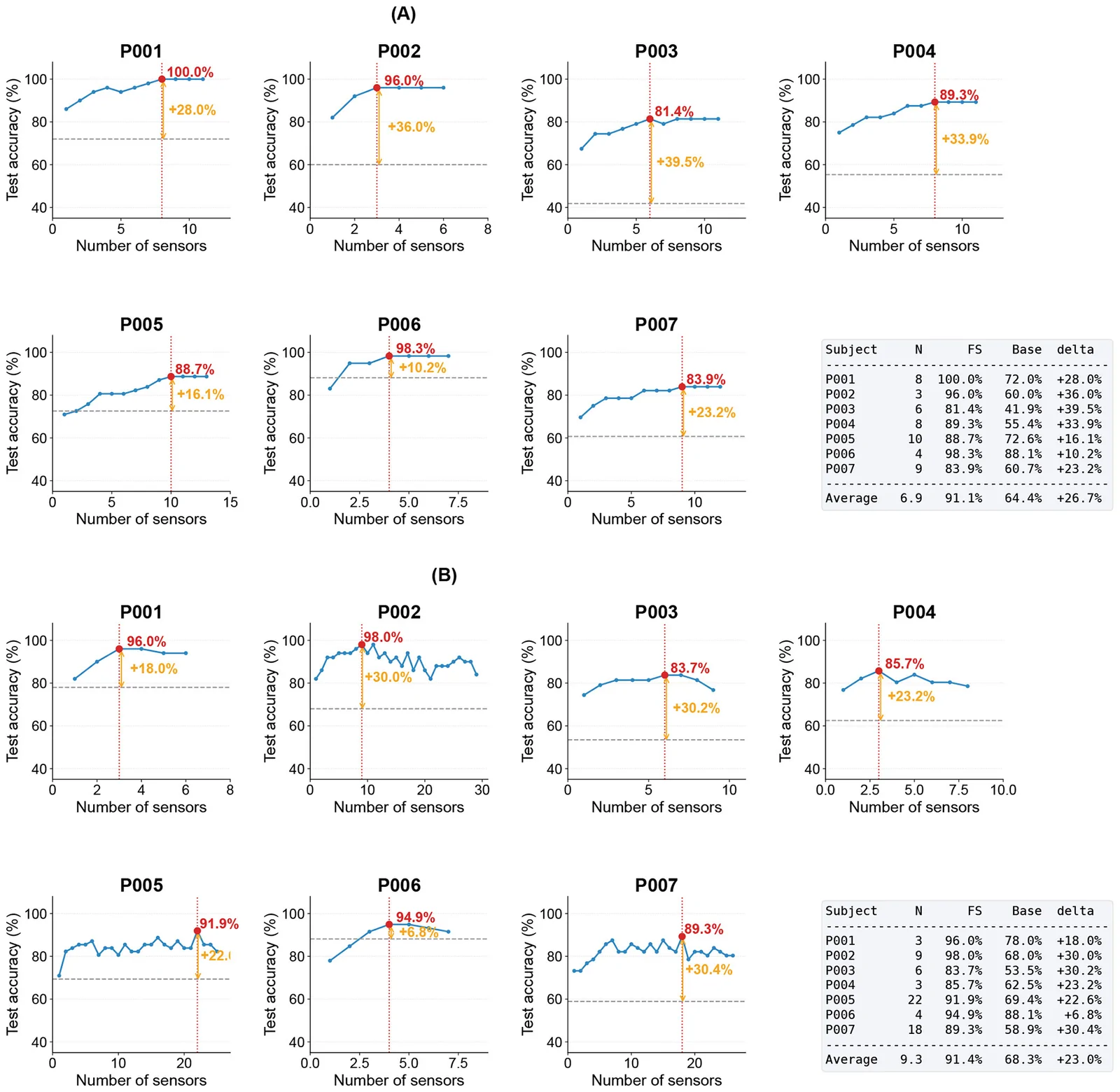
Forward sensor selection accuracy curves for **overt speech** [**(A)** support vector machine, **(B)** random forest]. Layout matches, Figure 4. The horizontal dashed line indicates the baseline accuracy using all 203 sensors, and the vertical dotted line marks the optimal sensor count $S^{*}$ at which accuracy peaked.

**Figure 7:**
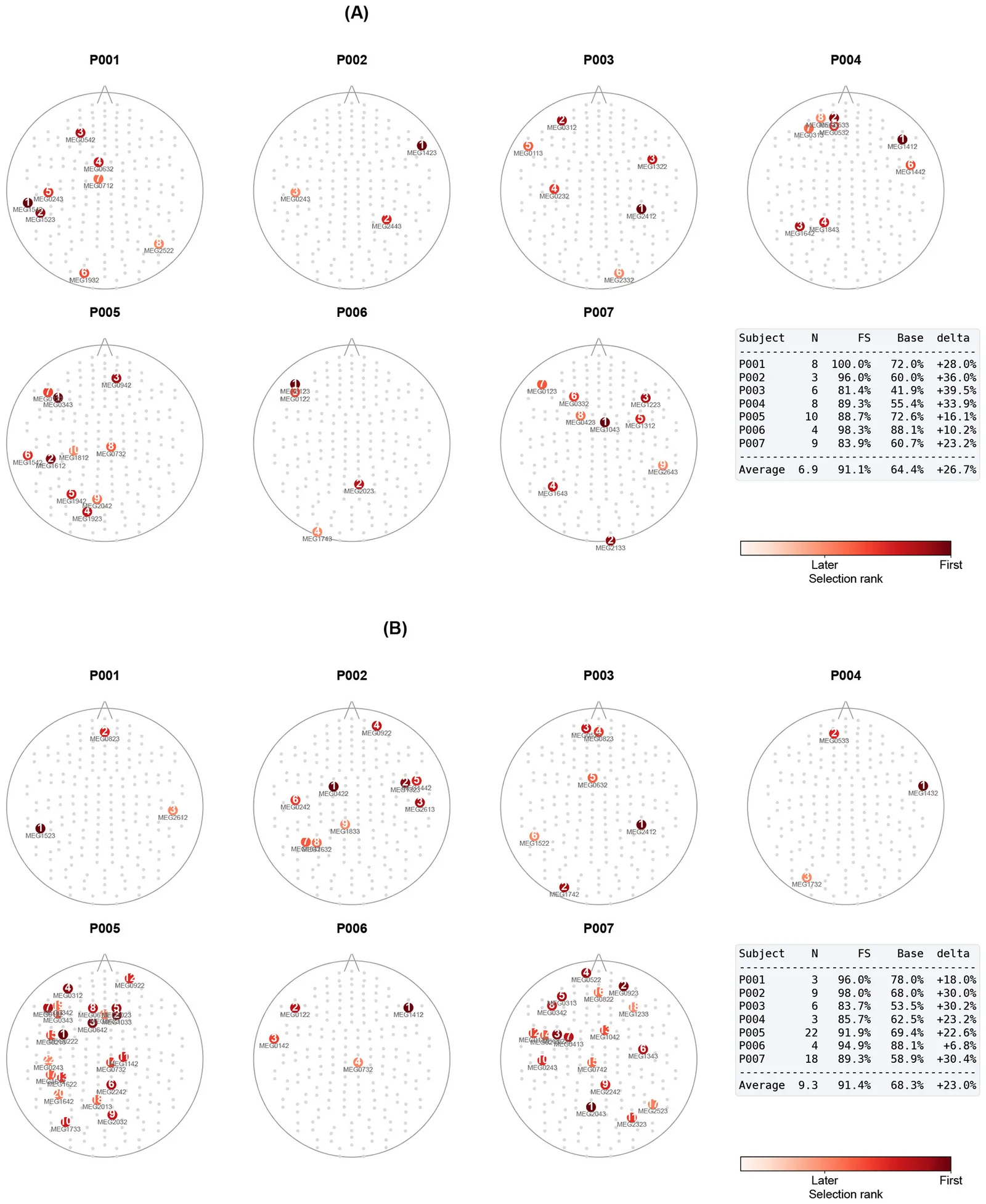
Optimal sensor locations for **overt speech** decoding using **(A)** support vector machines and **(B)** random forests. Layout matches Figure 5. Scalp topographies show each participant's optimal sensor subset $S^{*}$. Color indicates selection order (darker red, earlier selection; numbers denote rank). Gray dots indicate the full 203-channel montage.

### 3.2 Speech decoding using individual brainwaves from all sensors

Figure 8 shows the hold-out decoding accuracy for each of the seven DWT frequency bands, decoded independently using all 203 sensors, for both classifiers and speech phases. Across phases and classifiers, decoding accuracy varied substantially across frequency bands, indicating that distinct neural oscillatory components carry different amounts of information about spontaneous *yes*/*no* speech. Both the SVM and RF classifiers yielded consistent band rankings across participants. The Lower High-Gamma band achieved the highest average classification accuracy across participants for both intended and overt speech (65.6%±17.3% and 72.4%±17.2%, respectively, with SVM, and 64.1%±13.4% and 65.7%±14.5%, respectively, with Random Forest). In contrast, when using an SVM classifier, the Alpha band produced the lowest decoding performance.

**Figure 8:**
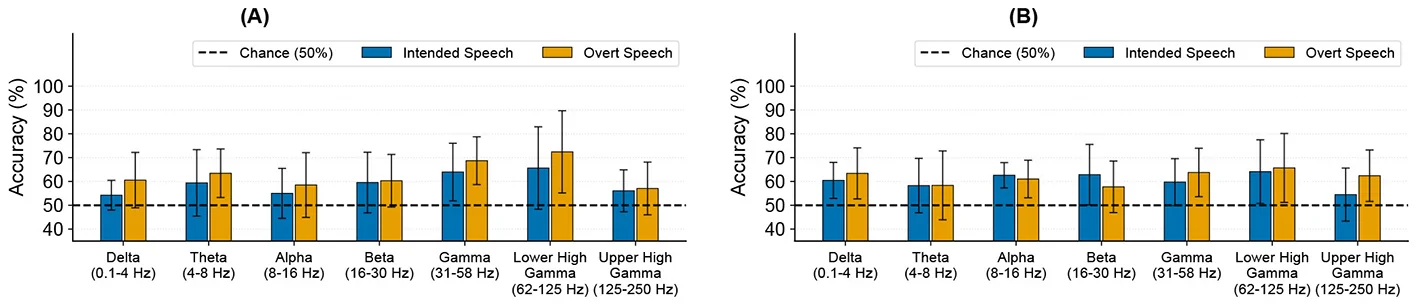
Individual brainwave decoding accuracy [**(A)** support vector machine; **(B)** random forest]. Classification accuracy (%) for each discrete wavelet transformed (DWT) frequency band decoded independently using all 203 sensors. Bands are ordered from highest to lowest frequency. Error bars indicate standard deviation across participants. The dashed line indicates the chance level (50%).

### 3.3 Speech decoding using individual brainwaves from the optimal sensor subset

Figure 9 shows decoding accuracy when each frequency band from the optimal sensor subset $S^{*}$ derived in Section 2.6.1 for each subject was used, rather than the full 203-channel montage. Overall, Lower High-Gamma still yielded slightly higher accuracies than other bands, together with the Beta band for intended speech. More importantly, those accuracies were overall lower than those using all bands (Figure 4 for both intended speech and Figure 6 for overt speech) or all frequency bands (Figure 8).

**Figure 9:**
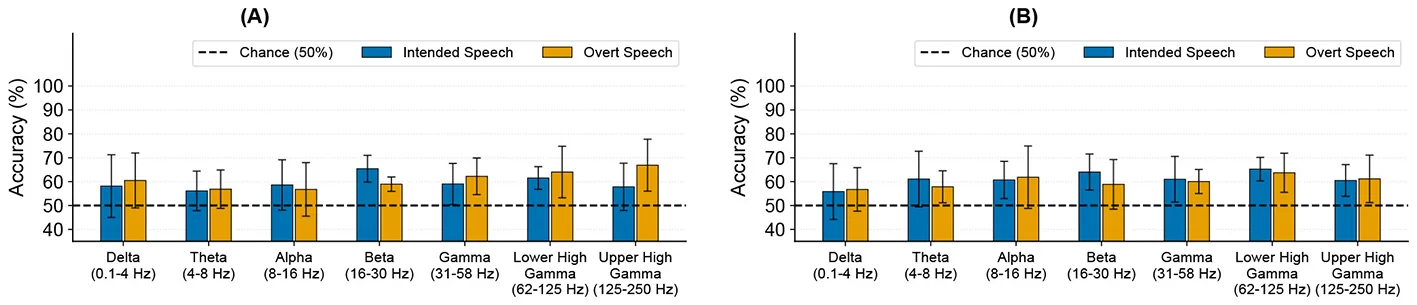
Individual band decoding accuracy with optimal sensor subset [**(A)** support vector machine; **(B)** random forest]. Classification accuracy (%) for each discrete wavelet transformed (DWT) frequency band decoded independently using the per-subject, phase-specific optimal sensor subset $S^{*}$ from forward selection (Section 2.6.1). Same layout as Figure 8. Error bars indicate standard deviation across participants. The dashed line indicates the chance level (50%).

We conducted a repeated-measures ANOVA with within-subject factors frequency band (7 levels) and speech segment (Intended/Overt), run separately for each classifier (SVM/RF), yielding four ANOVAs corresponding to the four panels in Figures 8, 9. These analyses were followed by Holm-corrected post-hoc paired t-tests comparing Lower High-Gamma to each of the other six bands.

The ANOVA results show that, for the SVM classifier using all sensors (Figure 8), band performance differs significantly, *F*_(6, 36)_ = 3.83, *p* = 0.0047. In contrast, the other three ANOVAs do not show significant effects of band: *F*_(6, 36)_ = 0.90, *p* = 0.502 for RF with all sensors (Figure 8, right), *F*_(6, 36)_ = 0.74, *p* = 0.624 for SVM with the optimal sensor set (Figure 9, left), and *F*_(6, 36)_ = 0.88, *p* = 0.517 for RF with the optimal sensor set (Figure 9, right).

*Post-hoc* analysis on SVM results in Figure 8 comparing Lower High-Gamma vs. each other band (Holm-corrected *t*-test, *N* = 7) showed that, for overt speech, Upper High-Gamma (72.4%) was significantly higher than Upper High-Gamma (57.1%) with a large effect [*t*_(6)_ = 5.35, *p* = 0.0017, Holm *p* = 0.0104, and *dz* = 2.02]. Also for overt speech, Lower High-Gamma vs. Delta/Theta/Alpha/Beta/Gamma all numerically favor Lower High-Gamma (+3.7 to +13.9 pts, *dz* = 0.44 − 0.94), but none survive Holm correction (all Holm *p*>0.24). For intended speech, the same direction was observed throughout (Lower High-Gamma numerically highest, dz up to 0.90), but nothing survives correction (all Holm *p*>0.33). In short, although there is a tendency for Lower High-Gamma to outperform other bands, statistical results show only one significance: Lower High-Gamma is significantly higher than Upper High-Gamma in overt speech decoding using SVM on all sensors. In addition, SVM (Figure 8) also showed a significant speech segment effect [*F*_(1, 6)_ = 10.15, *p* = 0.019], which indicates that overt speech accuracies are higher than intended speech accuracy overall. However, there was no significant band × speech segment interaction in any of the four ANOVA models.

## 4 Discussion

First, these findings in the baseline analysis verified the prior study (Dash et al., 2024) that spontaneous, intended and overt speech can be effectively decoded using non-invasive neuroimaging (i.e., MEG) signals, which provides a solid foundation for the neural decoding of uncued/spontaneous speech. These results also provide a foundation/baseline (Figure 3) for the core analysis of this study, including finding an optimal sensor set for this application, decoding the performance using individual brain waves from all sensors, and decoding the performance using individual brain waves for the optimal sensor set.

Second, our analysis of the optimal sensor subset suggests that the full set of more than 200 MEG sensors is unnecessary for this task. On average, only 7–8 sensors were sufficient to achieve decoding accuracies (85% to 91%) higher than those obtained using the full sensor set (60% to 71%). The improvement of accuracy was about 20% overall. We believe it is because the classifiers are too simple to handle the higher dimension when using the full set (203 sensors). This finding has critical implications for future practical applications, as the cost of the BCI system can be substantially reduced due to the smaller number of sensors. Our results also revealed considerable inter-subject variability in the optimal sensor subset, which ranged from 3 to 22. Five out of the seven participants obtained high decoding accuracy using fewer than 10 sensors, whereas the remaining two needed more in both intended and overt speech decoding. Current wearable OPM-MEGs are designed using a modular design regarding the number of sensors (e.g., 8, 16, 32, 64, and 128 sensors). With our findings, the system with the lowest cost could be achieved with just 24 sensors, making the overall cost much lower than that of full-scalp systems.

The spatial distribution of the selected sensors also shows cross-speaker variability while suggesting a level of consistency (Figure 5 for intended speech and Figure 7 for overt speech). For both classifiers (SVM and random forest), informative sensors clustered and showed a level of overlap across speakers, consistent with the involvement of cortical areas during speech preparation and production (e.g., motor cortex). However, there is notable variability between participants in the exact sensor locations, indicating that a personalized sensor setup may be ideal for practical application. In addition, there was no source localization analysis; our findings can be interpreted as a data-driven sensor subset, rather than exact locations to place sensors.

Third, in the individual brainwave band analysis, we observed each band obtained above-chance level accuracy using all sensors (Figure 8) or the optimal sensor set (Figure 9). These findings suggest that both intended and overt speech have a larger frequency range of brain oscillation (at least from 1 to 250 Hz), rather than being limited to one or a few bands. This finding is consistent with previous findings on overt and imagined speech (Dash et al., 2021b).

When comparing the performance between individual bands, Lower High-Gamma occasionally shows a higher numeric mean accuracy than other bands (i.e., Delta, Theta, Alpha, Beta, and Upper High-Gamma) in most settings (all sensors vs. optimal set, intended speech vs. over speech; Figures 8, 9). This is generally consistent with the literature and can be explained by the fact that the Lower High-Gamma band contains the primary cognition and semantic information (Dash et al., 2021b). However, statistical tests revealed there is no significant difference (possibly due to the smaller sample size, *N* = 7); thus, we would not conclude that Lower High-Gamma is the best individual band. Dash et al. (2021b) also had an interesting finding: the Delta band obtained high accuracy, which is probably due to the stimuli being short phrases, where the jaw motion is about the Delta frequency (about 4–5 Hz). In the current study, participants produced only short words (*yes*/*no*); there, the simple jaw motion may not contribute much information compared to phrases.

Although, we do not conclude that Lower High-Gamma is the best individual band, the observation or tendency we observe (Figures 8, 9) may suggest that Lower High-Gamma (up to 125 Hz) is an important band (at least more important than Upper High-Gamma) that a future wearable MEG-based BCI should provide, pending replication in a larger cohort. This requires a sampling rate of 250 Hz according to the Nyquist-Shannon sampling theorem (Shannon, 1949). In short, we nonetheless consider 250 Hz a reasonable practical target for wearable MEG sampling rates, pending replication in a larger cohort. Although current OPM systems can sample at rates of up to several kilohertz, current NV-diamond magnetometers (Sekiguchi et al., 2024) are limited to approximately 100 Hz, which would preclude resolving even the Lower High-Gamma band under this target. Of course, this suggestion does not mean that NV-diamond magnetometers should give up efforts to further improve sampling rates once they reach 250 Hz. It would be even better if a higher sampling rate (e.g., 500 Hz) could be provided.

Daily use of a whole helmet sensor montage may not be practical or cost-effective. These sensor selection results obtained from conventional whole-head SQUID-MEG systems provide a principled basis for designing sparse, wearable OPM- or NV-diamond-based magnetometer arrays for speech decoding. Unlike SQUID-MEG, OPM and NV-diamond magnetometers provide flexibility about the number of sensors and their locations. Our results suggest that future applications of real-world speech BCI devices would require an initial assessment to inform the placement of a reduced set of flexible sensors (e.g., 24 spatially distributed channels) in a wearable system for each user/patient, which would reduce the footprint of the wearable device. This transfer assumes that the spatial pattern of source activity underlying speech decoding is consistent between the three modalities and that a user's informative sensor locations remain stable across sessions and over time. Another limitation that can be explored is that head movement and day-to-day variability in sensor placement in wearable systems can introduce spatial misalignment relative to the original SQUID-derived map. Additionally, brain networks have been shown to be non-stationary and change over time due to plasticity, adaptive changes, and other factors (Ma et al., 2023; Silversmith et al., 2021; Wilson et al., 2026). As such, a one-time sensor selection assessment may need to be periodically revalidated to ensure that the transferred configuration remains optimal for reliable, long-term speech BCI use.

Finally, in all of these analyses across this study, the two classifiers (SVM and RF) obtained comparable performances, which suggests there is no model bias here. These performance levels (accuracies) are convincing and can be generalized. In other words, using a more powerful model, more advanced features, and/or by involving other techniques (such as dimension selection or reduction), the performance should be even better or at least not worse.

There are a few limitations in this study. The major limitation in this study is the relatively small sample size (number of participants, *N* = 7), which limits the robustness and generalization of these findings. Second, there is a small decoding set (vocabulary). While the design of this preliminary study was to establish decoding for truly spontaneously generated speech, a binary decoding set (*yes*/*no*) is far from the complexity of daily communicative speech. To establish a true understanding of the variability in optimal individual sensory selection, testing more subjects with more complex speech tasks is necessary. Nonetheless, we believe the current results provide insights into potential wearable MEG-based BCI design.

## 5 Conclusions

This study provided a foundation and addressed some design questions for the next-generation, wearable MEG-based BCI, toward practical communication. The neural speech decoding focused on the classification of a limited set of functional words rather than open-vocabulary decoding. Considering that the actual use of the BCI would be by patients (in a locked-in state) and for daily conversation, the decoding paradigm focuses on spontaneous, intended speech rather than cued speech, although overt speech decoding is provided for reference. Using a forward selection algorithm, the findings suggest that a small set of sensors ranging from 3 to 22 (average seven to eight) would be sufficient to obtain performance comparable to many sensors. Due to the modular design in the current OPM design, a set of 24 sensors would be sufficient for this specific task (yes/no decoding). The spatial distribution of these sensors shares a level of overlap across participants, but there is significant variability. A personalized sensor montage may be needed for each user/patient in real-world use, and the montage may need to be rechecked or updated periodically. Moreover, we analyzed the sampling frequency requirement by performing decoding using individual frequency bands. We observed that Lower High-Gamma (62–125 Hz) is an important band, even though it is not significantly higher than other bands (possibly due to our small sample size). Thus, we still suggest that at least a 250 Hz sampling frequency should be provided by future wearable MEG systems for speech decoding applications. However, we should be open to even higher sampling rates (e.g., 500 Hz), if possible. We recognize that current OPM systems can obtain rates of up to thousands of Hertz, however, the NV-diamond systems do not and could increase their sampling rate (and sensitivity) for practical communication.

## Data Availability

The raw data supporting the conclusions of this article will be made available by the authors, without undue reservation.

## References

[B1] Anumanchipalli G. K. Chartier J. Chang E. F. (2019). Speech synthesis from neural decoding of spoken sentences. Nature 568, 493–498. doi: 10.1038/s41586-019-1119-1 31019317 PMC9714519

[B2] Barrese J. C. Rao N. Paroo K. Triebwasser C. Vargas-Irwin C. Franquemont L. . (2013). Failure mode analysis of silicon-based intracortical microelectrode arrays in non-human primates. J. Neural Eng. 10:066014. doi: 10.1088/1741-2560/10/6/066014 24216311 PMC4868924

[B3] Beach S. D. Ozernov-Palchik O. May S. C. Centanni T. M. Gabrieli J. D. E. Pantazis D. (2021). Neural decoding reveals concurrent phonemic and subphonemic representations of speech across tasks. Neurobiol. Lang. 2, 254–279. doi: 10.1162/nol_a_00034 34396148 PMC8360503

[B4] Boto E. Holmes N. Leggett J. Roberts G. Shah V. Meyer S. S. . (2018). Moving magnetoencephalography towards real-world applications with a wearable system. Nature 555, 657–661. doi: 10.1038/nature26147 29562238 PMC6063354

[B5] Boto E. Meyer S. S. Shah V. Alem O. Knappe S. Kruger P. . (2017). A new generation of magnetoencephalography: room temperature measurements using optically-pumped magnetometers. Neuroimage 149, 404–414. doi: 10.1016/j.neuroimage.2017.01.034 28131890 PMC5562927

[B6] Card N. S. Wairagkar M. Iacobacci C. Hou X. Singer-Clark T. Willett F. R. . (2024). An accurate and rapidly calibrating speech neuroprosthesis. N. Engl. J. Med. 391, 609–618. doi: 10.1056/NEJMoa2314132 39141853 PMC11328962

[B7] Chi X. Hagedorn J. B. Schoonover D. D'Zmura M. (2011). EEG-based discrimination of imagined speech phonemes. Int. J. Bioelectromagn. 13, 201–206. Available online at: https://www.semanticscholar.org/paper/EEG-Based-Discrimination-of-Imagined-Speech-Chi-Hagedorn/c9794f1ca19866eae5f468ca971ec195d16bf225

[B8] Cohen D. Cuffin B. (1983). Demonstration of useful differences between magnetoencephalogram and electroencephalogram. Electroencephalogr. Clin. Neurophysiol. 56, 38–51. doi: 10.1016/0013-4694(83)90005-6 6190632

[B9] Dash D. Ferrari P. Berstis K. Wang J. (2021a). “Imagined, intended, and spoken speech envelope synthesis from neuromagnetic signals,” in Speech and Computer, eds. A. Karpov and R. Potapovapages (Cham: Springer International Publishing), 134–145. doi: 10.1007/978-3-030-87802-3_13

[B10] Dash D. Ferrari P. Malik S. Wang J. (2018). “Overt speech retrieval from neuromagnetic signals using wavelets and artificial neural networks,” in 2018 IEEE global conference on signal and information processing (GlobalSIP) (Anaheim, CA), 489–493. doi: 10.1109/GlobalSIP.2018.8646401

[B11] Dash D. Ferrari P. Wang J. (2020a). Decoding imagined and spoken phrases from non-invasive neural (MEG) signals. Front. Neurosci. 14:290. doi: 10.3389/fnins.2020.00290 32317917 PMC7154084

[B12] Dash D. Ferrari P. Wang J. (2020b). “Decoding speech evoked jaw motion from non-invasive neuromagnetic oscillations,” in 2020 international joint conference on neural networks (IJCNN) (Glasgow), 1–8. doi: 10.1109/IJCNN48605.2020.9207448

[B13] Dash D. Ferrari P. Wang J. (2021b). “Role of brainwaves in neural speech decoding,” in 2020 28th European Signal Processing Conference (EUSIPCO) (Amsterdam), 1357–1361. doi: 10.23919/Eusipco47968.2020.9287714

[B14] Dash D. Ferrari P. Wang J. (2024). Neural decoding of spontaneous overt and intended speech. J. Speech Lang. Hear. Res. 67, 4216–4225. doi: 10.1044/2024_JSLHR-24-00046 39106199 PMC12379578

[B15] Dash D. Wisler A. Ferrari P. Davenport E. M. Maldjian J. Wang J. (2020c). MEG sensor selection for neural speech decoding. IEEE Access 8, 182320–182337. doi: 10.1109/ACCESS.2020.3028831 PMC7668411 33204579

[B16] de Lange P. Boto E. Holmes N. Hill R. M. Bowtell R. Wens V. . (2021). Measuring the cortical tracking of speech with optically-pumped magnetometers. Neuroimage 233:117969. doi: 10.1016/j.neuroimage.2021.117969 33744453

[B17] Derix J. Iljina O. Weiske J. Schulze-Bonhage A. Aertsen A. Ball T. (2014). From speech to thought: the neuronal basis of cognitive units in non-experimental, real-life communication investigated using ECoG. Front. Hum. Neurosci. 8:383. doi: 10.3389/fnhum.2014.00383 24982625 PMC4056309

[B18] D'Zmura M. Deng S. Lappas T. Thorpe S. Srinivasan R. (2009). “Toward EEG sensing of imagined speech,” in Human-Computer Interaction. New Trends, ed. J. A. Jacko (Berlin; Heidelberg: Springer Berlin Heidelberg), 40–48. doi: 10.1007/978-3-642-02574-7_5

[B19] Ferrari M. Quaresima V. (2012). A brief review on the history of human functional near-infrared spectroscopy (fNIRS) development and fields of application. Neuroimage 63, 921–935. doi: 10.1016/j.neuroimage.2012.03.049 22510258

[B20] Garcia-Salinas J. S. Villaseor-Pineda L. Reyes-Garcia C. A. Torres-Garcia A. A. (2019). Transfer learning in imagined speech EEG-based BCIs. Biomed. Signal Process. Control 50, 151–157. doi: 10.1016/j.bspc.2019.01.006

[B21] Gramfort A. Luessi M. Larson E. Engemann D. A. Strohmeier D. Brodbeck C. . (2013). MEG and EEG data analysis with MNE-Python. Front. Neurosci. 7:267. doi: 10.3389/fnins.2013.00267 PMC3872725 24431986

[B22] Haresh M. V. Kannadasan K. Shameedha Begum B. (2025). An EEG-based imagined speech recognition using CSP-TP feature fusion for enhanced bci communication. Behav. Brain Res. 493:115652. doi: 10.1016/j.bbr.2025.115652 40482972

[B23] Herff C. Schultz T. (2016). Automatic speech recognition from neural signals: a focused review. Front. Neurosci. 10:429. doi: 10.3389/fnins.2016.00429 PMC5037201 27729844

[B24] Hughes C. L. Flesher S. N. Weiss J. M. Downey J. E. Boninger M. Collinger J. L. . (2021). Neural stimulation and recording performance in human sensorimotor cortex over 1500 days. J. Neural Eng. 18:045012. doi: 10.1088/1741-2552/ac18ad 34320481 PMC8500669

[B25] Iivanainen J. Carter T. R. Trumbo M. C. S. McKay J. Taulu S. Wang J. . (2023). Single-trial classification of evoked responses to auditory tones using OPM- and SQUID-MEG. J. Neural Eng. 20:056032. doi: 10.1088/1741-2552/acfcd9 37748476 PMC13333261

[B26] Iljina O. Derix J. Schirrmeister R. T. Schulze-Bonhage A. Auer P. Aertsen A. . (2017). Neurolinguistic and machine-learning perspectives on direct speech BCIs for restoration of naturalistic communication. Brain Comput. Interfaces 4, 186–199. doi: 10.1080/2326263X.2017.1330611

[B27] Kanas V. G. Mporas I. Benz H. L. Sgarbas K. N. Bezerianos A. Crone N. E. (2014). Joint spatial-spectral feature space clustering for speech activity detection from ECoG signals. IEEE Trans. Biomed. Eng. 61, 1241–1250. doi: 10.1109/TBME.2014.2298897 PMC4005607 24658248

[B28] Kohnen R. F. Lavrijsen J. C. M. Bor J. H. J. Koopmans R. T. C. M. (2013). The prevalence and characteristics of patients with classic locked-in syndrome in Dutch nursing homes. J. Neurol. 260, 1527–1534. doi: 10.1007/s00415-012-6821-y 23306659

[B29] Kundu S. Ari S. (2022). Brain-computer interface speller system for alternative communication: a review. IRBM 43, 317–324. doi: 10.1016/j.irbm.2021.07.001

[B30] Laureys S. Pellas F. Van Eeckhout P. Ghorbel S. Schnakers C. Perrin F. . (2005). The locked-in syndrome : what is it like to be conscious but paralyzed and voiceless? Prog. Brain Res. 150, 495–611. doi: 10.1016/S0079-6123(05)50034-7 16186044

[B31] Lee G. R. Gommers R. Wasilewski F. Wohlfahrt K. O'Leary A. (2019). PyWavelets: a Python package for wavelet analysis. J. Open Source Softw. 4:1237. doi: 10.21105/joss.01237

[B32] Ma X. Rizzoglio F. Bodkin K. L. Perreault E. Miller L. E. Kennedy A. (2023). Using adversarial networks to extend brain computer interface decoding accuracy over time. eLife 12:e84296. doi: 10.7554/eLife.84296 PMC10446822 37610305

[B33] Memarian N. Ferrari P. Macdonald M. J. Cheyne D. De Nil L. F. Pang E. W. (2012). Cortical activity during speech and non-speech oromotor tasks: a magnetoencephalography (MEG) study. Neurosci. Lett. 527, 34–39. doi: 10.1016/j.neulet.2012.08.030 PMC4884082 22926020

[B34] Moses D. A. Leonard M. K. Makin J. G. Chang E. F. (2019). Real-time decoding of question-and-answer speech dialogue using human cortical activity. Nat. Commun. 10:3096. doi: 10.1038/s41467-019-10994-4 PMC6667454 31363096

[B35] Moses D. A. Metzger S. L. Liu J. R. Anumanchipalli G. K. Makin J. G. Sun P. F. . (2021). Neuroprosthesis for decoding speech in a paralyzed person with anarthria. N. Engl. J. Med. 385, 217–227. doi: 10.1056/NEJMoa2027540 34260835 PMC8972947

[B36] Panachakel J. T. Ramakrishnan A. G. (2021). Decoding covert speech from EEG-A comprehensive review. Front. Neurosci. 15:642251. doi: 10.3389/fnins.2021.642251 PMC8116487 33994922

[B37] Pedregosa F. Varoquaux G. Gramfort A. Michel V. Thirion B. Grisel O. . (2011). Scikit-learn: machine learning in Python. J. Mach. Learn. Res. 12, 2825–2830. doi: 10.5555/1953048.2078195

[B38] Ramsey N. Salari E. Aarnoutse E. Vansteensel M. Bleichner M. Freudenburg Z. (2018). Decoding spoken phonemes from sensorimotor cortex with high-density ECoG grids. Neuroimage 180, 301–311. doi: 10.1016/j.neuroimage.2017.10.011 PMC6433278 28993231

[B39] Rezeika A. Benda M. Stawicki P. Gembler F. Saboor A. Volosyak I. (2018). Brain-computer interface spellers: a review. Brain Sci. 8:57. doi: 10.3390/brainsci8040057 29601538 PMC5924393

[B40] Roberts G. Holmes N. Alexander N. Boto E. Leggett J. Hill R. M. . (2019). Towards OPM-MEG in a virtual reality environment. Neuroimage 199, 408–417. doi: 10.1016/j.neuroimage.2019.06.010 PMC8276767 31173906

[B41] Sakai R. Kai A. Nakagawa S. (2021). “Classification of imagined and heard speech using amplitude spectrum and relative phase of EEG,” in 2021 IEEE 3rd global conference on life sciences and technologies (LifeTech) (Nara), 373–375. doi: 10.1109/LifeTech52111.2021.9391883

[B42] Scarapicchia V. Brown C. Mayo C. Gawryluk J. R. (2017). Functional magnetic resonance imaging and functional near-infrared spectroscopy: insights from combined recording studies. Front. Hum. Neurosci. 11:419. doi: 10.3389/fnhum.2017.00419 28867998 PMC5563305

[B43] Sekiguchi N. Fushimi M. Yoshimura A. Shinei C. Miyakawa M. Taniguchi T. . (2024). Diamond quantum magnetometer with dc sensitivity of sub-10 pT Hz^−1/2^ toward measurement of biomagnetic field. Phys. Rev. Appl. 21:064010. doi: 10.1103/PhysRevApplied.21.064010

[B44] Shannon C. E. (1949). Communication in the presence of noise. Proc. IRE 37, 10–21. doi: 10.1109/JRPROC.1949.232969

[B45] Silversmith D. B. Abiri R. Hardy N. F. Natraj N. Tu-Chan A. Chang E. F. . (2021). Plug-and-play control of a brain-computer interface through neural map stabilization. Nat. Biotechnol. 39, 326–335. doi: 10.1038/s41587-020-0662-5 32895549

[B46] Smith E. Delargy M. (2005). Locked-in syndrome. BMJ 330, 406–409. doi: 10.1136/bmj.330.7488.406 15718541 PMC549115

[B47] Song C. Xu R. Hong B. (2014). “Decoding of Chinese phoneme clusters using ECoG,” in 2014 36th annual international conference of the IEEE engineering in medicine and biology society (Chicago, IL), 1278–1281. doi: 10.1109/EMBC.2014.6943831 25570199

[B48] Suyuncheva A. Saada D. Gavrilenko Y. Schevchenko A. Vartanov A. Ilyushin E. (2021). “Reconstruction of words, syllables, and phonemes of internal speech by EEG activity,” in Advances in Cognitive Research, Artificial Intelligence and Neuroinformatics, eds. B. M. Velichkovsky, P. M. Balaban, and V. L. Ushakov, V. L. (Cham: Springer International Publishing), 319–328. doi: 10.1007/978-3-030-71637-0_37

[B49] Tang J. LeBel A. Jain S. Huth A. G. (2023). Semantic reconstruction of continuous language from non-invasive brain recordings. Nat. Neurosci. 26, 858–866. doi: 10.1038/s41593-023-01304-9 PMC11304553 37127759

[B50] Towle V. L. Yoon H.-A. Castelle M. Edgar J. C. Biassou N. M. Frim D. M. . (2008). ECoG gamma activity during a language task: differentiating expressive and receptive speech areas. Brain 131, 2013–2027. doi: 10.1093/brain/awn147 PMC2724904 18669510

[B51] Vansteensel M. J. Leinders S. Branco M. P. Crone N. E. Denison T. Freudenburg Z. V. . (2024). Longevity of a brain-computer interface for amyotrophic lateral sclerosis. N. Engl. J. Med. 391, 619–626. doi: 10.1056/NEJMoa2314598 39141854 PMC11395392

[B52] Wilson G. H. Stein E. A. Kamdar F. Avansino D. T. Pun T. K. Gross R. . (2026). Long-term unsupervised recalibration of cursor-based intracortical brain-computer interfaces using a hidden Markov model. Nat. Biomed. Eng. 10, 1466–1484. doi: 10.1038/s41551-025-01536-z PMC13375652 41361599

